# Fast Docking on Graphics Processing Units via Ray-Casting

**DOI:** 10.1371/journal.pone.0070661

**Published:** 2013-08-16

**Authors:** Karen R. Khar, Lukasz Goldschmidt, John Karanicolas

**Affiliations:** 1 Center for Bioinformatics, University of Kansas, Lawrence, Kansas, United States of America; 2 UCLA-DOE Institute for Genomics and Proteomics, University of California, Los Angeles, California, United States of America; 3 Department of Molecular Biosciences, University of Kansas, Lawrence, Kansas, United States of America; University of South Florida College of Medicine, United States of America

## Abstract

Docking Approach using Ray Casting (DARC) is structure-based computational method for carrying out virtual screening by docking small-molecules into protein surface pockets. In a complementary study we find that DARC can be used to identify known inhibitors from large sets of decoy compounds, and can identify new compounds that are active in biochemical assays. Here, we describe our adaptation of DARC for use on Graphics Processing Units (GPUs), leading to a speedup of approximately 27-fold in typical-use cases over the corresponding calculations carried out using a CPU alone. This dramatic speedup of DARC will enable screening larger compound libraries, screening with more conformations of each compound, and including multiple receptor conformations when screening. We anticipate that all three of these enhanced approaches, which now become tractable, will lead to improved screening results.

## Introduction

There are a number of structure-based methods for predicting small molecules that bind to specific sites on protein surfaces, most commonly active sites, intended for finding lead compounds in drug discovery efforts [1]. High throughput docking tools for “virtual screening” aim to dock thousands of compounds and predict several that will exhibit measurable binding, as a starting point for further optimization. This computational approach can have potential advantages over complementary “wetlab” screening methods because it can be less expensive and time consuming [1]. If successful, hits from a computational structure-based screen may also provide insights that guide the subsequent medicinal chemistry optimization in directions that would not be evident from the chemical structure of the hit compound alone.

Atomistic molecular dynamics simulations and detailed docking approaches are too computationally expensive to allow their direct use for many thousands of independent ligands, as required for most virtual screening applications [2]. Accordingly, several methods have been developed to speed up docking. Some entail using a reduced representation of the receptor, thus reducing the number of calculations associated with each energy evaluation [3]–[6]. Most approaches fix the receptor conformation or allow only limited conformational changes during docking, to reduce the number of degrees of freedom associated with the search [7]–[11]. While some methods allow the ligand conformation to vary during docking [9], [12], [13], others carry out independent docking trajectories using a series of pre-built low-energy ligand conformations (“conformers”) [7], [14], [15].

We have developed a docking tool called “Docking Approach using Ray Casting” (DARC), as part of the Rosetta macromolecular modeling software suite [16]. Our approach entails casting a set of rays from the protein center of mass to a series of points mapping out a surface pocket, thus building up a description of the topography of the protein surface as viewed from the protein interior. Since a complementary small-molecule bound to this site should have a complementary topography, we then cast the same set of rays towards the candidate inhibitor. If the inhibitor is indeed complementary to the protein surface, the intersection distance of each ray with the inhibitor should closely match the distance at which the ray reaches the protein surface. In a separate study we find that DARC proves capable of identifying known inhibitors from among large sets of decoy compounds, and we use DARC to identify new compounds active in biochemical assays against the anti-apoptotic protein Mcl-1 (manuscript in preparation: Gowthaman R, Miller S, Johnson D, Karanicolas J).

Despite using low resolution scoring and a fast minimization method (both are described in detail below), DARC screening nonetheless remained limited by computational restrictions. Our initial deployment of DARC to screen against Mcl-1 entailed screening only 12,800 compounds (with a maximum of 100 pre-built conformers per compound), and required 152,500 CPU hours to complete this screen. We found that we could achieve a speedup of approximately 6-fold by efficiently neglecting to calculate interactions of rays guaranteed not to contribute to the total score (the “ray elimination” step described later), but DARC remained limited by the size of compounds libraries that could feasibly be screened.

Graphics processing units (GPUs) were originally designed to process parallel, multithreaded 3D graphics via ray tracing, and have since evolved hardware to enable broader types of high throughput processes. Modern GPUs can process mathematical operations, support flow control, and have floating point precision. New libraries such as Compute Unified Device Architecture (CUDA, www.nvidia.com) and Open Computing Language (OpenCL, www.khronos.org/opencl) allow development of non-graphics programs for GPUs. These enable an application running on a central processing unit (CPU) to farm out parts of the job to a GPU. A variety of biomolecular modeling tasks have been adapted for GPU processing, from carrying out quantum calculations to calculating electrostatic surface potentials to stochastic modeling of chemical kinetics and molecular dynamics [17]–[22]. GPU computing has also been used to speed up certain other structure-based docking tools [23]–[29].

Given that the ray-casting step underlying our approach is highly analogous to the problem for which GPUs were originally developed, we reasoned that DARC would be highly amenable for porting to GPUs. Since each ray is scored separately and their scores are independent of one another, scoring is intrinsically a parallel process. Here we describe our adaptation of DARC for GPU scoring, leading to a speedup of approximately 27-fold over the corresponding calculation on a CPU alone.

## Methods

### Virtual screening using DARC

**Figure 1:**
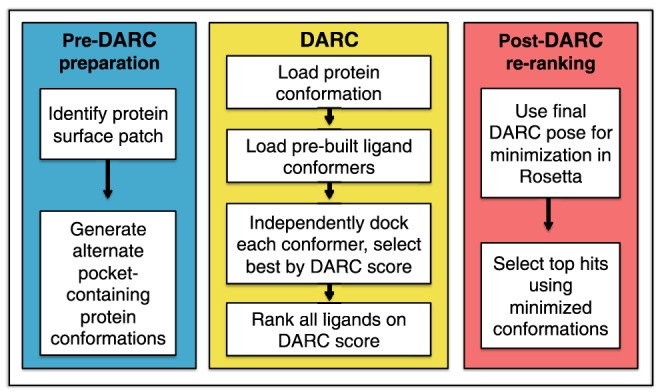
Docking overview. A schematic diagram of the complete workflow split into three stages: pre-DARC preparation, DARC, and post-DARC re-ranking.

An overview of the intended DARC workflow for virtual screening is diagrammed in **Figure 1**. The flow is separated into pre-DARC, DARC, and post-DARC stages.

In the pre-DARC preparation stage, a target pocket on the protein is identified and protein structures are generated for use with DARC. DARC was designed for docking at shallow pockets characteristic of those used by small-molecule inhibitors of protein-protein interfaces [30], [31]. The protein conformation is not moved during docking, and can come either from an experimental derived structure or from simulations designed to generate energetically favorable structures with diverse surface pocket shapes at the target site [32].

Each of these protein conformations is then used as a starting point for docking in DARC. Briefly, DARC sequentially carries out rigid body docking for each ligand conformer using a scoring function that maximizes the complementarity of the pocket and ligand shapes when viewed from the protein interior; the following two sections will describe the DARC scoring scheme and optimization protocol in detail. DARC is used to select the optimal conformer and docked pose for every member of the compound library.

The top-scoring model complexes (typically the best 10%) serve as a starting point for further optimization using the all-atom forcefield in Rosetta. This final energy minimization includes all rotatable dihedral angles (in both the protein and the ligand) as degrees of freedom. Finally, these minimized complexes are re-ranked on the basis of energetic considerations (e.g. interaction energy) as well as structural considerations (e.g. number of buried unsatisfied polar groups). The top scoring compounds can then be advanced for further characterization in biochemical or cell-based assays.

Since DARC scoring considers solely shape complementarity, the intended use of DARC is *not* as a standalone tool for predicting binding free energies, or even for predicting whether any particular compound is likely to bind the target protein. Rather, DARC is intended to provide a fast, low-resolution tool for identifying the likely binding mode of a compound. Our intended workflow thus separates the extensive burden of sampling (carried out by DARC using a crude scoring scheme) from the requirement of a detailed energy function to discriminate active from inactive compounds. This approach is in contrast to complementary methods such as RosettaLigand [33]–[35], which carries out detailed flexible-ligand docking via Monte Carlo simulations using the all-atom Rosetta energy function but is too computationally expensive to enable routine screening of large compound libraries.

### Scoring with DARC

DARC starts from a PDB file of a protein conformation, either from an experimentally derived structure or from biased “pocket optimization” simulations [32]. The shape of a surface pocket is defined using a grid-based method described in detail elsewhere [32]. Briefly, a grid is placed over the protein surface of interest. Based on the coordinates and radii of the atoms comprising the protein, grid points are marked either “protein” (P) or “solvent” (S). Solvent points which lie on a line between two protein points are then marked as “pocket” (to denote concave regions on the protein surface); this approach was originally used in the LigASite software [36].

**Figure 2:**
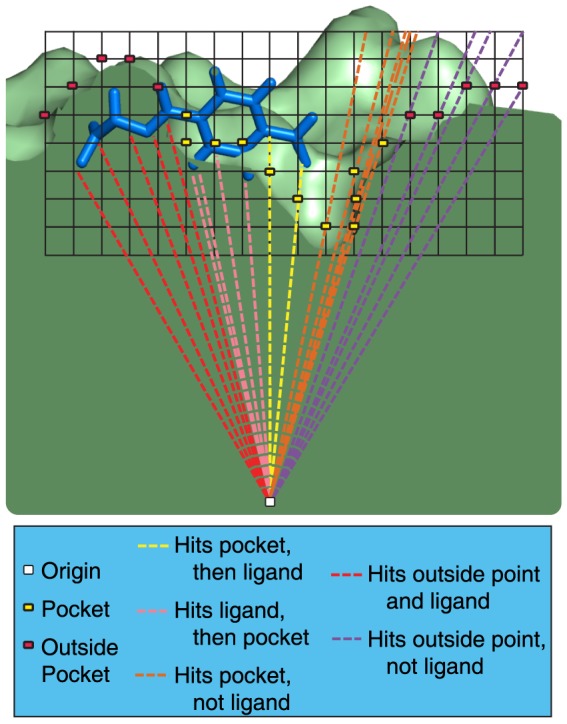
Docking approach using ray casting. A schematic diagram of DARC scoring is shown in cross section. A grid is placed at a region of interest on a protein surface, and used to identify “deep pocket” points. Points that are not in direct contact with the protein surface are removed, leaving behind a set of points that map the topography of the protein surface pocket (*yellow squares*). An adjacent layer of points on the protein surface are then labeled “forbidden” points (*red squares*). Rays are cast from an origin point within the protein (*white square*) at each pocket point and forbidden point. To score a docked pose, the same rays are cast at the ligand (*blue*), and the first intersection (if any) is calculated. The contribution to the total score from each ray is dependent on whether the ray was defined based on a pocket point or a forbidden point, and whether the ray intersects this point before or after it intersects with the ligand. These conditions are described in detail in the main text.

The pocket “shell” is identified as those pocket grid points in direct contact with the protein (**Figure 2**, *yellow squares*). Additional grid points are then added around the perimeter of the pocket shell (**Figure 2**, *red squares*), used to mark regions outside the pocket where ligand binding will not lead to favorable interactions (“forbidden” points). The direction from the pocket center of mass to the protein center of mass is defined, and a point 30 Å along this direction is defined as the origin from which rays will emanate (**Figure 2**, *white point*).

The angles and the distances expressing each of the shell points and forbidden points in spherical coordinates (relative to the origin point) are calculated and saved. The number of shell points and “forbidden” points that define the pocket – and thus the number of rays – depends both the grid spacing (typically 0.5 Å) and on the size of the surface pocket. In a typical use case, approximately 7,000 rays are used to define the protein pocket. This collection of vectors (representing points on this small region of the protein surface expressed in spherical coordinates) serves as a mapping of the protein surface topography that should be complemented by a well-docked ligand; the protein conformation and grid points are not directly used in docking beyond this point.

Given the position and orientation of a ligand to be scored, a series of rays are cast from the origin along each of the directions used to map the surface topography. For each ray, the distance at which the first intersection with the ligand occurs is calculated and subtracted from the (stored) distance at which the same ray hit the protein surface (i.e. the shell point). Each ray contributes to the total score as follows (where c1, c2, c3, and c4 are constants set to 1.0, 1.4, 21.6, and 9.5 respectively):


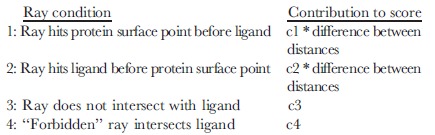


A highly complementary ligand will fill the pocket on the protein surface exactly; each contribution to the score represents some imperfection. A ray that hits the protein surface point before the ligand (condition #1) indicates unpacking in this docked pose (**Figure 2**, *yellow rays*). Conversely, a ray that hits the ligand before the protein surface (condition #2) indicates a steric clash (**Figure 2**, *pink rays*). A ray that does not intersect the ligand (condition #3) indicates that the ligand does not fully fill the surface pocket (**Figure 2**, *orange rays*), and “forbidden” rays that intersect the ligand (condition #4) indicate that the ligand extends beyond the boundaries of the surface pocket (**Figure 2**, *red rays*). Forbidden rays that do not intersect with the ligand do not contribute to the score (**Figure 2**, *purple rays*). The score assigned to the docked pose is taken as the sum of contributions from individual rays, divided by the number of contributing rays.

This approach to scoring is notably different from commonly-used docking tools, each of which estimate energies as the sum of contributions from interacting atom-atom pairs [1].

### Docking with DARC

Using this method for scoring poses, docking is then carried out using the particle swarm optimization (PSO) scheme [37] to optimize this objective function. Much like a genetic algorithm, this approach entails generating a set of candidate solutions (here called “particles,” each of which corresponds to a different docked pose). The position and orientation of each particle is then allowed to adapt in response to the other particles, moving towards the best-scoring local and global particles with a step size that depends on the relative scores of the particles [37]. After a number of iterations in which all particles move in response to one another, the “swarm” of particles ideally converges upon the globally optimal solution (in this case the lowest-scoring pose).

Though some docking approaches carry out sampling by greedy algorithms (such as incremental construction [38]), the most common approaches involve either individual Monte Carlo trajectories that sample Cartesian space or approaches that generate optimal solutions from a population of candidate solutions [1]. The latter class of methods, which include particle swarm optimization and genetic algorithms, make use of coupling between candidate solutions that can be advantageous in guiding the search towards optimal solutions: in the case of AutoDock, for example, a genetic algorithm was found to outperform a Monte Carlo simulated annealing protocol [39]. The potential drawback of this coupling lies in the fact that the inherent need for communication may preclude running candidate solutions on multiple separate machines. In the case of DARC (and virtual screening approaches that use genetic algorithms), however, the scoring function can be evaluated sufficiently rapidly that simulation of all candidate solutions (particles) can reasonably be evaluated on a single processor. Further, in a virtual screening context, running each member of the screening library as an independent job can still allow for parallelization across multiple machines.

In a typical use case, we generate ∼7,000 rays to map the protein pocket and dock ligands of ∼30 (non-hydrogen) atoms, iterating 200 times over a swarm comprised of 200 particles. This requires evaluating the DARC score for 40,000 docked poses, from a total of 8.4×10^9^ potential ray-atom intersections per simulation (210,000 potential ray-atom intersections per pose).

In practice, however, angular bounds can be computed from the docked pose that restrict which rays will intersect with a ligand. In other words, given a ligand atom radius and position relative to the origin, one can compute the maximum and minimum values of each angle required for intersection with this atom. Any rays that fall outside the bounds set by all atoms are guaranteed not to intersect with the ligand, and thus (in a step we call “ray elimination”) can be removed from consideration before this docked pose is scored. This reduces the number of ray-atom intersections that need to be computed, and leads to a speedup of about 6-fold when running on a CPU.

### DARC using GPU computing

As pointed out earlier, particles encoding the position and orientation of the ligand move collectively in response to one another, making this aspect of docking not naturally amenable to parallel computing. The scoring step, however, entails simultaneously evaluating the scores of 200 particles by summing independent contributions from a large number of rays; this represented a logical candidate for GPU computing.

DARC scoring was implemented on the GPU using the Open Computing Language (OpenCL), which allows the execution of custom programs called “kernels” on a variety of GPUs. Modern GPUs have hundreds of processing cores, thus allowing massive parallel execution of such kernels on a single GPU. Each kernel performs the same operation, but on a different data element from a large set. An important consideration for efficiently adapting DARC for GPU computing was avoiding latency associated with the cost of sending data between the CPU and the GPU.

**Figure 3:**
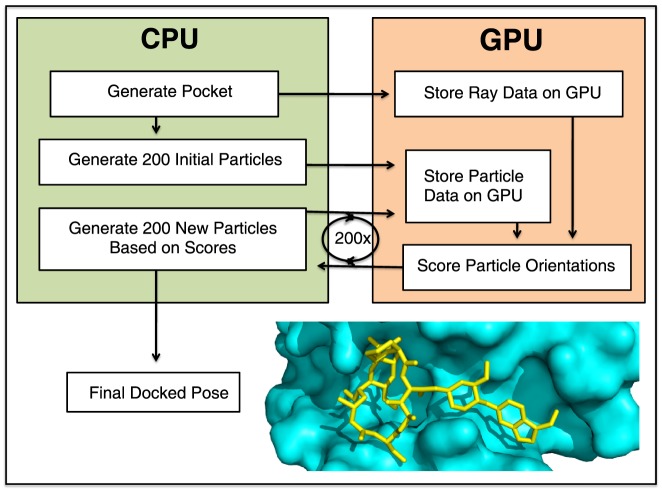
Control flow for GPU-enabled DARC. Control begins on the CPU. The CPU generates the pocket and casts rays at the protein surface, then stores this information on the GPU. The CPU generates 200 “particles” (independent initial ligand orientations to be used in the optimization) and passes each of these docked poses to the GPU. The GPU evaluates the DARC score of each docked pose, and passes these back to the CPU. The CPU uses these scores to update the docked poses accordingly, then sends the new poses to the GPU. This process is repeated 200 times, and the best-scoring particle is reported.

Our GPU implementation of DARC separates score evaluation (to be carried out on the GPU) from updating particle positions (to be carried out on the CPU) (**Figure 3**). We begin by storing information pertaining to rays (i.e. angles and the distance at which these hit the protein surface) on the GPU before optimization begins: this information will persist there, since it does not change over the course of the minimization. At each iteration of the optimization, information pertaining to *all* particles (i.e. ligand position and orientation) is transferred from the CPU to the GPU in a single step. The GPU uses a first kernel to compute the score contribution for a single ray to *every* particle. In the typical use case described above, each of 7,000 processes is therefore responsible for computing the potential intersection with the 6,000 atoms comprising the swarm (200 particles with 30 atoms each). A second kernel is then applied to each of the 200 particles, to sum the 7,000 contributions from each ray to the score of this particle. Through the use of the second kernel on the GPU, only 200 scores corresponding to particles must be returned to the CPU, instead of 1,400,000 scores from individual rays. Once the scores for each of the particles have been transferred, the CPU uses these scores to update the ligand position and orientation encoded by each particle accordingly.

### DARC PSO scoring on CPU and GPU

DARC scoring on a CPU occurs as follows:

*Loop over Particles {*

*Identify the max/min angles required for intersection with the ligand*

*Loop over Rays {*

*Check if Ray angles may allow intersection with ligand*

*If Ray may intersect with ligand {*

*Loop over Atoms in current Particle {*

*If Ray intersects Atom {*

*Calculate distance of first intersection*

*Save this distance if it is the lowest of all Atoms*

*}*

*}*

*}*

*Save the contribution of this Ray for the current Particle*

*}*

*Particle score* = *Sum of Ray scores/Number of Contributing Rays*

}

Scoring with the GPU version occurs using two separate two kernels. The first kernel processes one ray per thread as follows:

*Get rayID for this process, define current Ray*

*Loop over Particles {*

*Loop over Atoms in current Particle {*

*Calculate distance of first intersection with current Ray, if intersection occurs*

*Save this distance if it is the lowest of all Atoms in this Particle*

*}*

*Calculate the contribution of the Ray for the current Particle, store it on GPU*

}

The second kernel processes one particle per thread as follows:

*Get particleID for this process, define current Particle*

*Loop over Ray scores for this Particle {*

*Add to current score*

*}*

*Particle score* = *Sum of Ray scores/Number of Contributing Rays*

### Running DARC in Rosetta

DARC is implemented in the Rosetta software suite [16]. Calculations described here were carried out using svn revision 52964 of the developer trunk source code. Rosetta is freely available for academic use (www.rosettacommons.org), with the new features described here included in the 3.6 release.

The standard Rosetta can be built enabling GPU processing as follows (it may be necessary to alter rosetta_source/tools/build/basic.settings to add the address of individual OpenCL headers):

scons mode = release extras = opencl bin

Input files for small molecules are generated in two steps. The first involves downloading the ligand in the SMILES format from the ZINC database [40], then creating a pdb format file with multiple conformers with using the Omega software [41]–[43] as follows:

*OpenEye/bin/omega2 -in molecule.smi* –*out molecules.pdb* –*maxconfs #conformers*

When creating multiple conformers, they can be separated by babel as follows:

*babel* –*ipdb molecules.pdb* –*opdb molecule.pdb -m*

In the second step, a parameter file for the ligand is created with babel and the Rosetta python app molfile_to_params, as follows:

*babel* –*ipdb molecule.pdb* –*opdb molecule.sdf*

*molfile_to_params.py* –*c* –*nKHR* –*pmol molecule.sdf*

The Rosetta command line used to generate a set of rays (rays.txt) that define a protein pocket topography is as follows (for target residue number 105 of protein Bcl-xL with the file 2YXJ.pdb):

*make_rayfiles.linuxgccrelease* –*iinput_protein_file 2YXJ.pdb* –*central_relax_pdb_num 105*

The Rosetta command line used to run DARC on a GPU using these input files is as follows:

*DARC.opencl.linuxgccrelease* –*input_protein_file 2YXJ.pdb* –*input_ligand_file molecule.pdb* –*extra_res_fa molecule.params* –*eggshell_triplet rays.txt* –*gpu 1*

## Results

### Determining suitable stopping criteria

The two key parameters that determine the DARC runtime are the number of particles and the number of iterations. In order to determine the extent of sampling required for adequate convergence, we evaluated the difference in DARC score obtained from simulations of varying computational requirements against the score obtained from an intensive “gold-standard” simulation. As a model system, we randomly selected a compound from the ZINC database of commercially available compounds [40], ZINC00057615, and docked a single conformer of this compound to a pocket on the surface of the protein Bcl-xL (PDB ID 2yxj).

**Figure 4:**
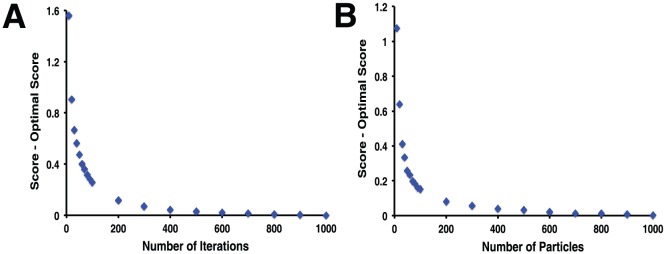
Effect of the number of particles and the number of iterations on DARC score. To determine the number of particles and number of iterations required for reasonable convergence of the DARC score, docking was carried out with (**A**) an increasing number of iterations while holding the number of particles fixed at 200, and (**B**) an increasing number of particles while holding the number of iterations fixed at 200. Differences in score are reported relative to the “gold standard,” taken to be the most extensive simulation in the set (i.e. 1000 iterations or 1000 particles).

We initially fixed the number of particles at 200, and sequentially extended the number of iterations from 10 up to our “gold standard” value of 1000 iterations. As expected, increasing the length of our trajectories led to progressively lower final scores (**Figure 4a**), at the expense of a linear increase in (CPU) runtime (not shown). While the docked score decreased rapidly at first, much of the improvement had already been realized after 200 iterations: extending the trajectory beyond this point led only to a modest decrease in score. For this reason, we adopted 200 iterations as our “typical use” value.

We then turned to the number of particles for inclusion, and carried out an analogous experiment. Using 200 iterations in all cases, we sequentially increased the number of particles from 10 up to our “gold standard” value of 1000 particles. As expected, increasing the number of particles similarly led to better solutions (**Figure 4b**), again with a linear increase in runtime (not shown). Based on the diminishing benefit of including a large number of particles, we adopted 200 particles as our “typical use” value.

**Figure 5:**
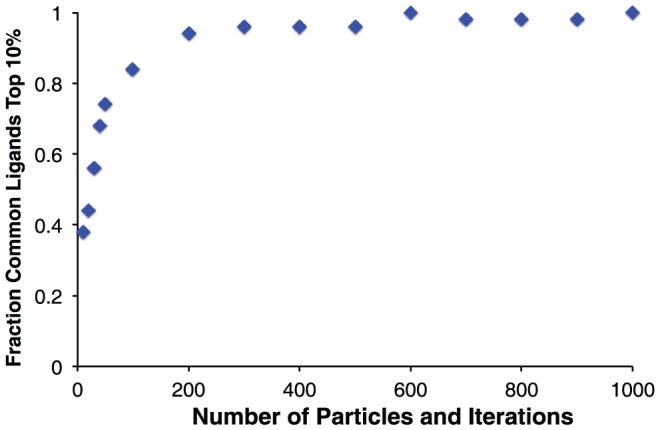
Effect of the number of particles and the number of iterations on the “hit” compounds selected. The most pragmatic measure of convergence is the identity of the “hits” to be advanced for further evaluation. The top scoring 10% of the compound library from the most extensive docking simulations were considered to be the “gold standard” hits. With increasing computationally intensive simulations (by together increasing the number of particles and the number of iterations), an increasing fraction of the hits are members of the “gold standard” set.

To put these results in the more pragmatic context of virtual screening experiment, we then compiled a set of 1000 randomly selected compound from the ZINC database [40], and evaluated how the extent of sampling would affect the ranking of these compounds against the same Bcl-xL surface pocket. We started with a “gold standard” ranking of each member of our library, by carrying out docking with DARC using 1000 particles and 1000 iterations. We marked the top-scoring 10% of the library (100 compounds) as “hits,” then asked how many of these “hit” compounds would remain in the top 10% if docking was carried out using a reduced number of iterations and particles. We found that 94 of the 100 hit compounds were recovered in the top-scoring 10% using our “typical use” parameters of 200 particles and 200 iterations (**Figure 5**), with little benefit associated with more extensive sampling. We therefore carried forward these values for the further studies described below.

### DARC speedup on Graphics Processing Units (GPUs)

All timing comparisons described below were carried out using a GeForce GTX 580 GPU, which can run 1024 threads concurrently, and a Dual Intel Xeon E5-2670 CPU using one thread.

As a first timing benchmark, we evaluated the time needed to carry out docking using the same model system described earlier: a single conformer of ZINC00057615 docked against a pocket on the surface of the protein Bcl-xL. Based on our typical grid spacing (0.5 Å) and the size of the surface pocket we would typically use about 7,000 rays to describe this pocket; for benchmarking, we instead reduced the grid spacing to generate 93,000 initial rays then varied the number of rays used in docking by generating subsets of this large collection.

As expected, the time required to complete this calculation scales approximately linearly with the number of rays and the number of particles, whether carried out entirely on a CPU (**Figure S1a**) or with the help of a GPU (**Figure S1b**). While the scaling is similar, however, the calculations are completed much more quickly using the GPU: in a typical uses case (7,000 rays, 200 particles and 200 iterations), the CPU takes 93 seconds to carry out the calculation and the GPU takes 3.4 seconds, corresponding to a 27-fold speedup (**Figure S1c**).

Similar behavior is observed when docking a single conformer to a surface pocket at the functional site of another protein, Mdm2 (**Figure S1d–f**). Due to the different size and shape of this pocket, the same grid spacing would lead to only 3,000 rays to describe this protein surface. Under these conditions (again with the standard 200 particles and 200 iterations), the calculation would take 47 seconds using the CPU alone, or 3.2 seconds using the GPU (a 15-fold speedup).

**Figure 6:**
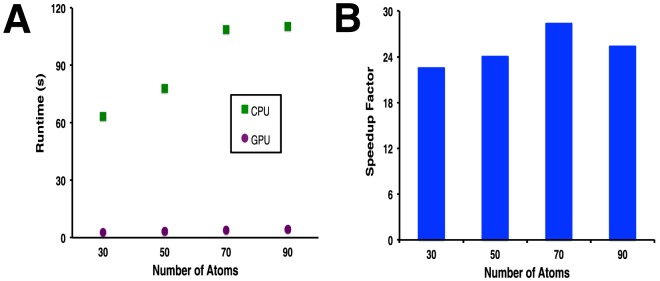
Dependence on number of atoms in the ligand. Ligands of varying sizes were docked using DARC. **A**) Time required to complete the optimization, using a CPU alone or with the GPU. **B**) Speedup factor, reported as the ratio of the time required using the GPU to the time required using the CPU alone.

We next tested the scaling of time with regards to the number of atoms in the ligand, docking to Mdm2 using 5,000 rays and 200 particles. We used a series of ligands containing 20 (ZINC0043625), 25 (ZINC00469420), 30 (ZINC01280234), 35 (ZINC01298436), and 40 (ZINC02091520) non-hydrogen atoms. We find that the time required for this calculation on the CPU alone is not linearly related to the number of ligand atoms (**Figure 6a**), because the geometry of the ligand dictates how much of the calculation can be avoided through the “ray elimination” step. In all cases, carrying out this calculation using the GPU results in a speedup of about 25-fold (**Figure 6b**).

**Figure 7:**
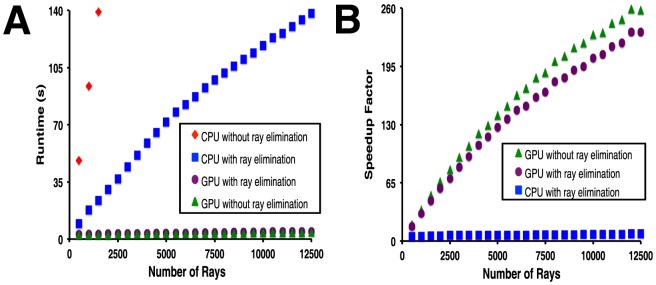
Comparison of DARC optimization with and without the “ray elimination” step. The “ray elimination” step is found to significantly improve performance of DARC on the CPU alone, but made little difference when the GPU is used. **A**) Time required to complete the optimization, using a CPU alone or with the GPU. **B**) Speedup factor, reported as the ratio of the time required using the GPU to the time required using the CPU alone.

While the typical-use speedup in the examples here is dramatic, we note that these data in fact *downplay* the true difference stemming from the use of the GPU for these calculations. In the timings we have reported above, the algorithm carried out on the CPU includes the “ray elimination” step that reduces the number of potential ray-atom intersections to be considered. The GPU calculations described above, however, do not include this step; we made a design decision not to take advantage of the potential for fewer calculations on the GPU, because the ray elimination step would cause threads to become asynchronous. This branch divergence in the kernel execution would lead to uncoalesced memory access, slowing the total time required for the calculation. For a straightforward comparison, we therefore additionally tested a variation of the CPU code that does not include the “ray elimination” step, and a variation of the GPU code that does include this step (**Figure 7**). We find that the GPU optimization requires a very similar time to reach completion regardless of whether or not the “ray elimination” step is used, justifying our design decision. As expected, the opposite holds for the CPU version: performance is significantly slower when the “ray elimination” step is not used. In a typical use case for Bcl-xL comprising 7,000 rays, the GPU version of DARC without the “ray elimination” step is completed about 180-fold faster than the same calculation on the CPU alone.

### Analysis and implications of DARC speedup on GPUs

**Figure 8:**
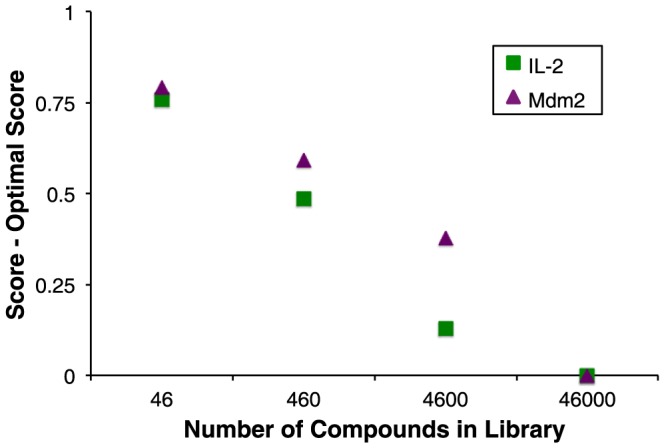
The GPU-enabled speedup facilitates screening of larger libraries, which in turn allows better-scoring ligands to be identified. Compound libraries of increasing size were screened against interleukin-2 and Mdm2. As expected, screening larger libraries led to identification of compounds with better scores. All scores are reported relative to the lowest scoring ligand in the largest set.

As described earlier, a key motivation in adapting DARC for GPU processing stemmed from the practical limitation on the size of compound libraries that can be routinely screened: our initial deployment of DARC entailed screening only 12,800 compounds, and required vast computational resources. To test whether extending our library size would improve the quality of compounds identified – subject to the DARC objective function – we carried out an experiment to determine the effect of library size on the resulting hit compounds. Since virtual screening involves drawing those few compounds from the extreme end of the distribution of scores, we trivially anticipated that increasing library size would lead to a monotonic improvement in the score of the top-scoring compound. Accordingly, we built a library of 46,000 compounds corresponding to a drug-like subset of the ZINC database [40], then used this to build further incrementally smaller libraries (decreasing the library size 10-fold each time). We carried out a virtual screen of each library against two protein targets, interleukin-2 (PDB ID 1m47) and Mdm2 (PDB ID 4jvr), and unsurprisingly observed a considerable decrease in the DARC score for the top-scoring compound as we increased our library size (**Figure 8**). These results serve to illustrate the fact that chemical space is not heavily covered by (random) compound libraries of this size, and that computational enhancements that enable screening of larger compound libraries are likely to enable identification of more optimal compounds for the target of interest – subject to the strong caveat that compounds with better scores may not necessarily show more activity, depending on the objective function.

**Figure 9:**
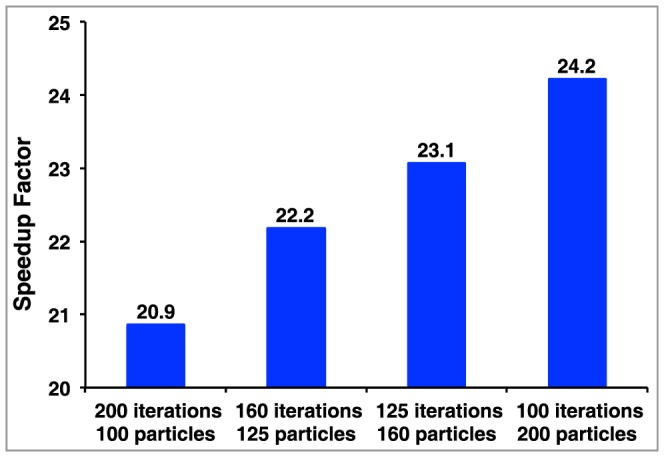
Runtime dependence on the number of particles and the number of iterations. A series of optimizations are compared in which the number of calculations (and thus the total time required) on the CPU is constant, and the speedup factor is reported as the ratio of the time required using the GPU to the time required using the CPU alone. The benefit of using the GPU is enhanced when individual GPU tasks are larger (more particles), allowing fewer CPU-GPU communication steps.

With an eye towards additional optimization of our GPU adaption of DARC in the future, we sought to better understand the rate-limiting step in our current implementation. Based on the relatively weak dependence of the GPU timing on factors that dictate the number of potential ray-atom intersections to be considered (number of rays, number of ligand atoms, and number of particles) (**Figure S1**), we surmised that GPU calculation itself was not the rate-limiting step in the overall calculation. To test this hypothesis, we carried out minimizations of Bcl-xL (with our typical use case of 7,000 rays), but varied the number of iterations while keeping the product of the number of iterations and the number of particles was fixed. As expected from fixing the total number of potential ray-atom intersections to be computed, the CPU alone required an almost identical amount of time to complete each of these calculations, confirming that calculating ray-atom intersections was indeed rate-limiting. If the same step was rate-limiting when carried using the GPU implementation, we would expect each of these calculations to again require a fixed amount of time for completion. In contrast, the use of the GPU allowed faster calculations upon decreasing the number of iterations but using more particles: this in turn lead to a greater overall speedup with respect to the CPU implementation (**Figure 9**). We further found that up to eight independent GPU-DARC threads running on eight (CPU) cores required the same time for completion as a single GPU-DARC thread, despite *sharing* a single GPU (not shown). Collectively these observations suggest that given a “typical use” setup in the current implementation, the portion of the calculation carried out on the GPU is *not* rate-limiting; rather the rate-limiting step lies either in the CPU-GPU communication step occurring once per iteration or, more likely given that a GPU can be effectively shared between multiple cores, lies in the few remaining calculations taking place on the CPU. The implications of these observations will be discussed further below.

## Discussion

Here we describe a faster implementation of the DARC ligand-docking program enabled by GPU computing. By carrying out the scoring step on GPUs, we achieve a speedup a 180-fold speedup over the same calculation carried out on a CPU alone. This calculation could be carried out 6-fold faster on the CPU by eliminating certain interactions from consideration before scoring, but this algorithmic difference did not affect timing on the GPU. Accordingly, the GPU-enabled code is therefore 27-fold faster than our fastest CPU-only code. This speedup was achieved using a modern GPU that is relatively inexpensive (less than $500).

Several other docking tools have recently been adapted to make use of GPU computing, leading to reported speedups in ranging from 2-fold to 100-fold (**Table 1**). Methods that require long serial trajectories, such as those built upon molecular dynamics [23], [24], require frequent CPU-GPU communication. This in turn leads to latency that limits the speedup achievable through GPU computing. A feature common to tools that achieve dramatic speedup is the ability to break up tasks into parallel subtasks that are either very numerous (i.e. DARC, PLANTS, GPUperTrAmber) or else individually computationally intensive (i.e. AutoDock Vina): either approach leads to long stretches of computing carried out exclusively on the GPU without the need for communication with the CPU. By extension, for applications such as DARC in which the objective function can be easily ported for calculation on the GPU, optimization schemes that simultaneously consider multiple candidate solutions (such as genetic algorithms and particle swarm optimization) are exceptionally well-suited to achieve dramatic speedups through relatively minor code changes.

**Table 1:** Comparison of GPU-enabled docking tools.

Docking tool	GPU enabled functionality	Speedup
Molecular dynamics combined with docking	Molecular dynamics	2–3× [23]
DOCK6	Amber scoring (molecular dynamics)	6.5× [24]
ZDOCK/PIPER/Hex	Fast Fourier Transforms	15× [25]
MolDock	Initially only scoring, then also differential evolution	27× [26]
*DARC*	*Simultaneously scoring multiple particles*	*27×*
PLANTS	Concurrent grid-based search	60× [27]
AutoDock Vina	Runs docking concurrently from different starting orientations	62× [28]
GPUperTrAmber	Scoring very large systems by decomposition	100× [29]

Docking methods have been adapted for GPU computing using a variety of strategies. These require different degrees of CPU-GPU communication, and accordingly enable varying speedups relative to the analogous CPU-only protocol.

In the case of our GPU-enabled DARC implementation, these insights provide inspiration by which further speedups may be possible. As noted earlier, the fact that all particles move collectively in response to one another does not make porting the entire PSO calculation to the GPU an attractive approach for achieving further speedup. However, the fact that eight CPU cores can share a single GPU without noticeable slowing implies that the GPU remains under-utilized in our current implementation; this in turn suggests that the current framework could be adapted by increasing the size of the problem allocated to the GPU at each iteration. Through further careful examination of the relationship between the number of particles and the number of iterations (**Figure 9**), it may prove possible to achieve equivalent convergence more quickly more particles and fewer iterations. Alternatively, further parallelization may be realized by bundling particles corresponding to different ligand conformers for simultaneous scoring on the GPU, rather than carry out separate (serial) optimization of each conformer. The fact that additional calculations can be likely carried out on the GPU with little additional cost also offers the opportunity to fundamentally change the DARC scoring paradigm: either by simultaneously using multiple sets of rays originating from distinct origins within the protein, and/or by adding new components to capture effects of electrostatics. In short, any enhancement that increases the computational burden per iteration that is carried by the GPU is likely to yield further speedup relative to the CPU alone.

Given fixed computational resources allocated for completion of a project, the ability to carry out docking more rapidly will have profound implications for applications of DARC. In the most obvious case, this speedup will allow screening against very large libraries that previously may not have been tractable, for example the complete ZINC database [40] or a library of hypothetical compounds likely amenable to straightforward synthesis [44]. Even in cases in which a relatively small library of interest is to be screened (for example, computational screening of a library of compounds currently available in-house), this speedup will allow an increase in the number of conformers screened per compound; this in turn is expected to reduce the number of false negatives in the screen, by increasing the likelihood of including an active conformer. This speedup may further allow the use of multiple pre-built receptor conformations for docking [45]–[51], providing a means to implicitly represent receptor flexibility and thus allow further diversity in collection of hits identified.

## Supporting Information

Figure S1 **Dependence of simulation time on number of rays.** A single ligand conformation was docked in the Bcl-xL (**A–C**) or Mdm2 (**D–F**) surface pocket, independently varying the number of rays defining the pocket and the number of particles. **A,D**) Time required to complete the optimization using a CPU. **B,E**) Time required to complete the optimization with the GPU. **C,F**) Speedup factor, reported as the ratio of the time required using the GPU to the time required using the CPU alone.
(EPS)
